# Strong sustained type I IFN signaling acts cell intrinsically to impair IFNγ responses and cause tuberculosis susceptibility

**DOI:** 10.64898/2026.01.19.700487

**Published:** 2026-01-22

**Authors:** Stefan A. Fattinger, Roberto A. Chavez, Kristen C. Witt, Bianca Parisi, Jesse J. Rodriguez, Elizabeth A. Turcotte, Ella C. Brydon, Marian R. Fairgrieve, Harmandeep Dhaliwal, Angus Y. Lee, Dmitri I. Kotov, Russell E. Vance

**Affiliations:** 1 Division of Immunology and Molecular Medicine, Department of Molecular and Cell Biology, University of California, Berkeley, CA, USA.; 2 Cancer Research Laboratory, University of California, Berkeley, CA, USA.; 3 Center for Emerging and Neglected Diseases, University of California, Berkeley, CA, USA; 4 Present address: Division of Infectious Diseases, Department of Medicine, Andrew M. and Jane M. Bursky Center for Human Immunology and Immunotherapy Programs, Washington University School of Medicine, St. Louis, MO, 63110, USA.; 5 Howard Hughes Medical Institute, University of California, Berkeley, CA, USA.

## Abstract

*Mycobacterium tuberculosis* (*Mtb*) causes over one million annual deaths, but most infected individuals never exhibit symptoms. Type I interferons (IFNs) have emerged as a major factor driving *Mtb* susceptibility, but how type I IFNs impair immunity to *Mtb* is a key unresolved question. Here we show that an early and primary effect of type I IFN during *Mtb* infection is the cell-intrinsic impairment of IFNγ signaling. IFNγ signaling was selectively impaired in the subset of infected macrophages experiencing high and sustained levels of type I IFN signaling. Genetic elimination of RESIST, a recently described positive regulator of type I IFN production, specifically eliminated the high and sustained type I IFN response, fully restored IFNγ signaling, and rescued *Mtb* susceptibility without affecting basal type I IFN responses. Our results demonstrate that strong and sustained type I IFN responses specifically and cell-intrinsically impair responsiveness to IFNγ to cause *Mtb* susceptibility.

## Introduction

Tuberculosis (TB) is caused by *Mycobacterium tuberculosis* (*Mtb*) and is the deadliest infectious disease of humans, with over one million yearly deaths world-wide^1^. The standard treatment for TB involves a 4–6 month course of a combination of antibiotics that is often poorly tolerated and is also ineffective against increasingly prevalent multi-drug resistant *Mtb*. *Mtb* infection elicits a robust innate and adaptive immune response that protects most but not all infected individuals from disease. A major unresolved question is how variations in the immune response to *Mtb* result in severe disease in some individuals. Interferon-γ (IFNγ) has been implicated in protection against mycobacterial infections in humans^2–5^, and is essential in mice for resistance to *Mtb*^6–10^. However, most *Mtb*-susceptible humans and mice exhibit robust IFNγ production, indicating that IFNγ is often insufficient for protection. Indeed, BCG and a recent vaccine candidate that induce IFNγ-production by *Mtb*-specific T cells nevertheless fail to protect against infection in adults^11–15^. In addition, IFNγ is also not always necessary for protection against *Mtb*^16–20^. The factors that control the necessity and sufficiency of IFNγ for the control of tuberculosis remain poorly understood.

In contrast to IFNγ, type I IFNs—including IFNα, IFNβ, and other isoforms, all of which signal through the interferon-α/β receptor (IFNAR)—are clearly associated with progression of *Mtb* disease in humans^21–25^. A host-detrimental role of type I IFNs during *Mtb* infection has also been observed in mice in diverse experimental contexts^26–35^. Type I IFNs similarly impair resistance to other intracellular bacterial pathogens, including *Listeria monocytogenes* and *Legionella pneumophila*, though the precise mechanisms by which type I IFNs broadly impair anti-bacterial immunity remain unclear. A consistent finding is that type I IFN signaling impairs IL-1-mediated immunity by various mechanisms, including by modulating lipid mediator production, or by the upregulation of IL-1 receptor antagonist (IL-1Ra) or IL-10^34,36,37^. However, type I IFN signaling can promote susceptibility even in the absence of IL-1 signaling^37^, and IL-1Ra-deficiency also rescues mouse models lacking type I IFN-driven susceptibility^38^. Thus, existing data suggest that additional mechanisms, beyond suppression of IL-1, may contribute to type I IFN–driven susceptibility^39,40^.

Recently, we identified SP140 as an important negative regulator of type I IFN responses^41,42^. *Sp140*-deficient mice (on a C57BL/6J (B6) genetic background) exhibit increased production of type I IFNs compared to isogenic wild-type mice, resulting in uncontrolled *Mtb* replication, hypoxic granulomas, and an exacerbated inflammatory phenotype similar to that seen in humans with clinical TB^32,41^. Importantly, the *Mtb* susceptibility of *Sp140*^−/−^ mice is rescued by crosses to *Ifnar*^−/−^ mice. Thus, *Sp140*^−/−^ mice are a useful genetic model for the mechanistic dissection of type I IFN-driven tuberculosis disease. Importantly, *Ifnar*-deficiency does not greatly impact the *Mtb* susceptibility of wild-type B6 mice implying that the basal levels of type I IFNs seen in B6 mice are not pathogenic during *Mtb* infection^34,36,43–45^. A major outstanding question is how elevated, but not basal, levels of type I IFN specifically impair immunity to *Mtb*.

SP140 is a transcriptional repressor that we recently showed specifically represses two tandemly duplicated and nearly identical genes, *Resist1* and *Resist2*, both of which encode the identical protein RESIST (REgulated Stimulator of Interferon via Stabilization of Transcript)^42^. *Sp140*^−/−^ macrophages express higher levels of RESIST, which we found stabilizes *Ifnb1* mRNAs by specifically impairing the activity of the CCR4-NOT polyA-tail dead-enylase to promote *Ifnb1* mRNA turnover^42^. *Resist1/2*-deficiency eliminates the elevated levels of *Ifnb1* mRNA and IFNβ protein seen in *Sp140*^−/−^ mice, and returns IFNβ expression to the basal levels seen in B6 mice^42^, but whether this also restores resistance to *Mtb* remains unknown.

In the lungs of humans and mice, intracellular *Mtb* replication occurs mainly in interstitial macrophages (IMs), a type of myeloid cell in which T cells are only inconsistently able to mediate *Mtb* control^46,47^. Cell-type specific deletion of *Ifnar* on CD64^+^ myeloid cells rescues the susceptibility of *Sp140*^−/−^ mice^32^, implying that type I IFNs act on myeloid cells, but it remains unclear whether type I IFNs act cell intrinsically on infected macrophages to impair control of *Mtb* replication, or instead act cell extrinsically, e.g., by inducing macrophage production of cytokines such as IL-10 to suppress responses by other cells. Since type I IFNs impair *Mtb* restriction, and T-cells employ IFNγ to restrict *Mtb*^10,16,48,49^, we hypothesized that inconsistent IFNγ-mediated control of *Mtb* might arise from differential exposure of infected macrophages to type I IFNs. In support of this hypothesis, it has been shown that type I IFN signaling can impair the induction of certain IFNγ-induced interferon stimulated genes (ISGs)^50–53^. Moreover, elevated type I IFN ISG induction correlated with decreased expression of IFNγ-induced ISGs and detrimental outcomes in human *Mycobacterium leprae* infections^54^. Similar negative correlations between type I IFN responses and IFNγ responses have also been observed in *Listeria*-infected mice^55^. During *Mtb* infection, *in vitro* experiments have shown that type I IFN can impair the induction of IL-12—a key proinflammatory cytokine that promotes IFNγ production—in a cell-extrinsic manner by inducing IL-10^56^. *In vivo*, we have recently shown that the enhanced type I IFN response seen in *Sp140*^−/−^ mice correlates with an attenuated IFNγ response in *Mtb*-infected interstitial macrophages^32^. However, it was not shown that type I IFNs caused the decreased responsiveness to IFNγ. In addition, these analyses were performed at day 25 post infection, when bacterial loads had already diverged, making it difficult to disentangle cause and effect. Moreover, there was no evidence that the cells exhibiting decreased IFNγ responsiveness were a susceptible niche for *Mtb* replication. Thus, it remains unclear if there is a causal relationship in which type I IFN signaling cell-intrinsically impairs the IFNγ response of infected macrophages *in vivo*, and whether such impairment causes *Mtb* susceptibility.

Here, we take advantage of *Sp140*^−/−^ mice to systematically dissect the emergence and cause of type I IFN–driven susceptibility to *Mtb*. We show that type I IFNs exert a broad and sustained impairment of IFNγ responses in primary macrophages. We establish CXCL9 and Viperin proteins as robust markers of IFNγ and type I IFN responses, respectively, and use these markers to show that type I IFNs suppress IFNγ responsiveness in IMs during *Mtb* infection. Importantly, impairment of IFNγ responsiveness is selective for the subset of macrophages experiencing strong responses to type I IFNs. This subset of macrophages also exhibits the highest burdens of *Mtb*. By generating mixed *Sp140*^−/−^ bone marrow chimeras containing IFNAR-proficient and -deficient cells, we further demonstrate that type I IFNs act cell-intrinsically to impair responsiveness to IFNγ. By crossing *Sp140*^−/−^ mice to a sensitive type I IFN reporter mouse line, we uncover a temporal progression in which type I IFN signaling precedes and subsequently diminishes IFNγ responsiveness, thereby rendering IMs a permissive intracellular niche for *Mtb*, leading to tuberculosis disease susceptibility. Finally, by genetic elimination of RESIST in *Sp140*^−/−^ mice, we demonstrate that sustained and strong (but not basal) type I IFN signaling specifically suppresses IFNγ responsiveness and drives *Mtb* susceptibility.

## Results

### IFNAR-STAT2-IRF9 signaling broadly impairs IFNγ signaling

**Figure 1.**
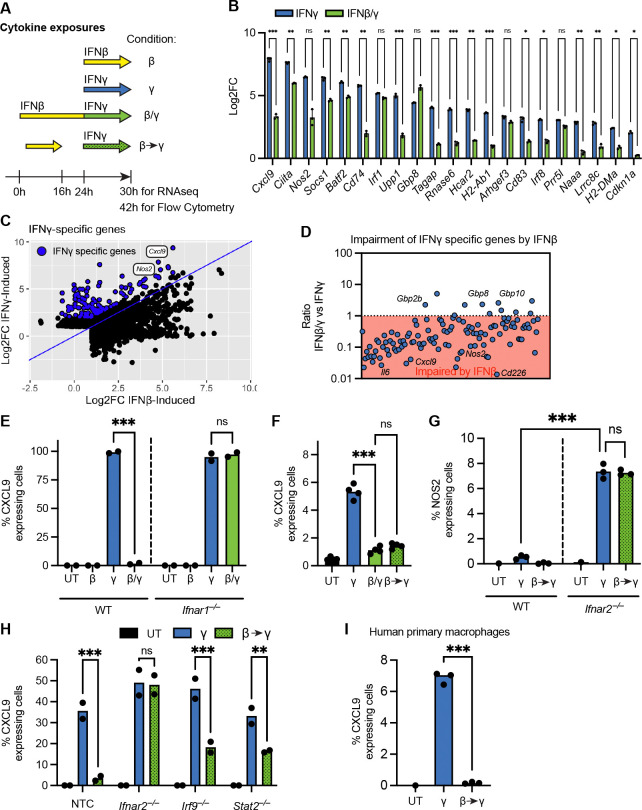
IFNAR-STAT2-IRF9 signaling broadly impairs IFNγ signaling. **(A)** Schematic of cytokine exposures for RNAseq and flow cytometry analysis. **(B)** Log2 fold change induction of previously defined IFNγ signature genes upon exposure to IFNγ with or without IFNγ preexposure. **(C)** Induction of IFN-specific genes upon IFNγ and IFNγ. In blue, all genes that are at least two-fold more induced upon IFNγ compared to IFNγ, which are defined as IFNγ specific genes. **(D)** Ratio comparing the induction of IFNγ specific genes upon IFNβ/γ vs IFNγ treatment. IFNγ induction is inhibited by IFNβ preexposure for genes with a ratio <1 (red area), whereas IFNγ induction is promoted by IFNβ preexposure for genes with a ratio >1. **(E)** Quantification of CXCL9 protein expressing bone narrow derived macrophages (BMMs) from B6 wild-type (WT, left half) or *Ifnar1*^−/−^ (right half) mice upon exposure to IFNβ, IFNγ or IFNβ/γ. UT = Untreated **(F)** Quantification of CXCL9-expressing BMMs to assess the effect of IFNβ pre-exposure, without (β/γ) or with (β→γ) its removal prior to IFNγ stimulation. **(G)** Quantification of NOS2 expressing BMMs from WT (left half) or *Ifnar2*^−/−^ (right half) mice upon IFNβ, IFNγ or IFNβ→γ. **(H)** Quantification of CXCL9-expressing BMMs deficient in IFNAR signaling components, IFNAR, IRF9, and STAT2. Gene disruption made with Cas9-RNPs. NTC = Non-Target Control. **(I)** In human primary macrophages, quantification of CXCL9-expressing cells upon exposure to IFNβ, IFNγ or IFNβ→γ. Data in (B-H) are from BMMs and in (I) from human primary macrophage. Statistical significance was calculated in (B) with RM two-way ANOVA with the Geisser-Greenhouse correction and Sidak’s multiple comparison test, in (E,G,H) with two-way ANOVA with Sidak’s multiple comparison test in (E,G) or Turkey’s multiple comparison test in (H), in (F,I) one-way ANOVA with Sidak’s multiple comparison test. *p < 0.05, **p < 0.01, ***p < 0.001, ns = not significant.

We hypothesized that type I IFNs act on infected macrophages to impair their responsiveness to IFNγ. To explore this hypothesis, we first tested whether type I IFN globally impairs the IFNγ-induced transcriptional response, or just affects certain IFNγ-induced genes as previously shown^50–53^. We exposed mouse bone marrow-derived macrophages (BMMs) to IFNβ (type I IFN) alone, IFNγ alone, or both cytokines simultaneously, and performed RNAseq (conditions referred to as β, γ and β/γ, respectively; see Figure 1A for exposure details). Most ISGs are similarly induced by IFNβ and IFNγ, but we previously identified a subset of 21 ISGs that are preferentially induced by IFNγ and that are upregulated during *Mtb* infection^32^ (referred to as IFNγ signature genes). Pre-exposing BMMs to IFNβ blunted IFNγ-induced expression of almost all IFNγ signature genes (Figure 1B). A notable exception was *Gbp8*, which was induced by IFNγ similarly in IFNβ-pretreated or control cells. To test if type I IFN signaling impairs the IFNγ response beyond these previously defined IFNγ signature genes, we defined a broader subset of 121 ISGs that are induced at least two-fold more by IFNγ as compared to IFNβ (Figure 1C). Strikingly, 91% (110/121) of all these ISGs showed reduced expression levels upon IFNγ when pre-exposed to IFNβ (Figure 1D). *Gbp8*, *Gbp2b* and *Gbp10* were among the 11 ISGs (Table S1A) that were induced by IFNγ despite IFNβ pretreatment.

The chemokine CXCL9 is a canonical IFNγ-induced ISG that is highly expressed during human and non-human primate *Mtb* infections, and has been suggested to promote an effective T cell mediated immune response^57,58^. In our RNAseq dataset, *Cxcl9* was the most IFNγ-upregulated gene and was highly preferentially induced by IFNγ versus IFNβ (>8 fold more induced by IFNγ than IFNβ, Figure 1C), a result that we validated by RT-qPCR (Figure S1B). We also performed intracellular staining and flow cytometry to determine if CXCL9 was specifically induced by IFNγ at the protein level (see Figure 1A, which includes exposure details for flow cytometry-based experiments). Strikingly, despite some induction of *Cxcl9* transcripts by IFNβ (Figure 1C), we only detected CXCL9 protein expression upon IFNγ exposure (Figures 1E, S1C). In line with the RNAseq results, IFNβ pre-exposure fully abolished the induction of CXCL9 protein (Figures 1E, S1C). As expected, IFNβ blockade of CXCL9 induction required the IFNβ receptor, IFNAR (Figures 1E, S1C). Importantly, neither a 10-fold increase in IFNγ concentration, nor co-stimulation with TNF or TLR-agonists, could overcome the IFNβ-mediated blockade of CXCL9 induction by IFNγ (Figures S1D–E). The impairment of IFNγ signaling persisted at least 8h after the removal of IFNβ, a timepoint at which type I IFN specific *Rsad2* transcripts (encoding Viperin) had already returned to baseline (Figures 1F, S1F, compare β/γ to β>γ, see Figure 1A for exposure details).

Nitric Oxide Synthase 2 (NOS2) is another IFNγ-induced ISG implicated in *Mtb* protection in mice^8,59–61^. In our RNAseq dataset, *Nos2* transcripts were also highly and specifically expressed upon IFNγ (Figure 1C). In sharp contrast to CXCL9, NOS2 was barely detectable at the protein level when cells were treated with IFNγ (Figure 1G) consistent with previous reports^62^. Strikingly, however, *Ifnar2*^−/−^ cells induced abundant NOS2 protein in response to IFNγ (Figure 1G). This result implies that tonic type I IFN-signaling is sufficient to impair IFNγ-induced NOS2 expression and explains the previously observed failure of IFNγ to induce NOS2 in wild-type cells. Unlike CXCL9, the impairment of NOS2 protein expression was partially reversed when cells were co-stimulated with TLR agonists in addition to IFNγ (Figure S1G). Given these observations, and the fact that the role of NOS2 in protection from TB in humans is still debated^63^, we chose CXCL9 as the most reliable and specific marker for the IFNγ response in subsequent analyses.

Signaling downstream of IFNAR involves the transcription factors STAT1, STAT2 and IRF9 that are believed to form a complex commonly called Interferon-stimulated gene factor 3 (ISGF3). ISGF3 binds specific DNA elements termed interferon-sensitive response element (ISREs) to induce ISG expression^64^. To determine whether these signal transducers are involved in the impairment of IFNγ responsiveness, we used CRISPR-Cas9 ribonuclear protein complexes (RNPs) to generate STAT2- and IRF9-deficient BMMs, and then exposed the knockout cells to IFNγ with or without IFNβ pre-exposure. Interestingly, both STAT2 and IRF9-deficency partially reversed type I IFN-driven inhibition, which suggest that both are important in their functions downstream of IFNAR signaling (Figure 1H).

Next, to confirm the relevance of our findings to human cells, we exposed human immortalized THP-1 cells and primary monocyte derived macrophages to IFNs. In both human-derived cell types, we could detect a robust inhibition of IFNγ-induced CXCL9 by type I IFN signaling, demonstrating that type I IFN exposure also impairs the IFNγ response in human macrophages (Figures 1I, S1H).

Together, these data, along with prior observations^50–53^, demonstrate that in mouse and human macrophages, type I IFN signaling downstream of IFNAR can engage STAT2 and IRF9 to broadly impair responsiveness to IFNγ in a robust and sustained manner.

### Type I IFNs impair IFNγ responses to *Mtb in vivo*

Correlative data from mycobacterial infected humans suggest that elevated type I IFN signaling accompanies an attenuated antibacterial IFNγ response^22,54^, consistent with observations in genetic mouse studies of *Listeria* and *Mtb* infections^43,53,55^. Moreover, we recently observed an attenuated IFNγ response in *Mtb*-infected *Sp140*^−/−^ mice that exhibit elevated type I IFN signaling^32^. However, a causal relationship between type I IFN signaling and attenuation of the IFNγ response, and whether such attenuation promotes susceptibility to *Mtb*, remains to be shown *in vivo*.

**Figure 2.**
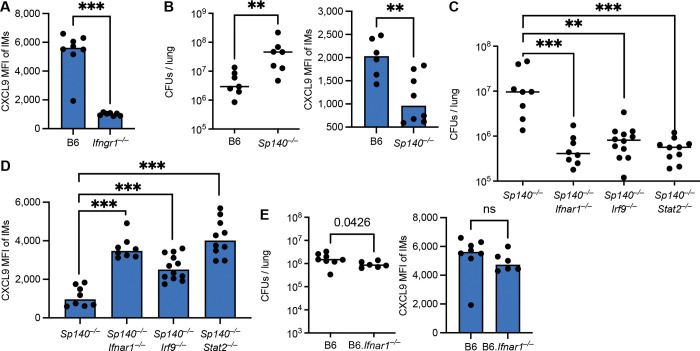
Type I IFNs impair the IFNγ response to *Mtb in vivo*. **(A)** Mean Fluorescence Intensity (MFI) of CXCL9 staining in interstitial macrophages (IMs) from *Mtb*-infected B6 and *Ifngr1*^−/−^ mice at day 25 post infection (pi). **(B)** Colony Forming Units (CFUs) per lung (left panel) and MFI of CXCL9 staining in IMs (right panel) from *Mtb*-infected B6 and type I IFN susceptible *Sp140*^−/−^ mice at day 25 pi. **(C)** CFUs per lung and **(D)** MFI of CXCL9 staining in IMs from *Mtb*-infected *Sp140*^−/−^, *Sp140*^−/−^*Ifnar1*^*−/−*^, *Sp140*^−/−^*Irf9*^−/−^ and *Sp140*^−/−^ *Stat2*^−/−^ mice at day 28–29 pi. **(E)** CFUs per lung (left panel) and MFI of CXCL9 staining in IMs (right panel) from *Mtb*-infected B6 and B6.*Ifnar1*^−/−^ mice at day 25–26 pi. Statistical significance was calculated for CFU data in (B, E) with Mann-Whitney test and in (C) Kruskal-Wallis test with Dunn’s multiple comparison test, for MFI data in (A,B,E) Welch’s t-test and in (D) Brown-Forsythe/Welch ANOVA test, both with Dunnett’s T3 multiple comparisons test. *p < 0.05, **p < 0.01, ***p < 0.001, ns = not significant.

Based on the *in vitro* results above, we sought to test whether we can use CXCL9 as a marker for IFNγ responsiveness on infected IMs during *Mtb* infection *in vivo*. We infected mice with a low aerosolized dose of *Mtb* Erdman strain (20–100 CFUs), and focused our analysis on IMs—the main intracellular niche for *Mtb* replication^47^ (see Figure S2A for gating strategy). At day 25 post infection (pi), we detected significantly lower levels of intracellular CXCL9 in *Ifngr1*^−/−^ mice (lacking the IFNγ receptor) as compared to wild-type B6 mice (Figure 2A), validating the use of CXCL9 as a reliable marker for the IFNγ response *in vivo*. Next, to test whether enhanced type I IFN signaling represses responsiveness to IFNγ *in vivo*, we infected B6 and *Sp140*-deficient mice. *Sp140*^−/−^ exhibit higher levels of type I IFN and heightened susceptibility of *Mtb* that is *Ifnar*-dependent^41^. IMs from *Sp140*^−/−^ mice expressed lower levels of CXCL9, indicative of reduced IFNγ responsiveness, as compared to B6 mice (Figure 2B). To test whether the decreased expression of CXCL9 observed in *Sp140*^−/−^ mice was due to IFNAR signaling, we examined *Sp140*^−/−^*Ifnar1*^−/−^ mice. As previously reported^41^, these mice are rescued from the *Mtb* susceptibility seen in *Sp140*^−/−^ mice (Figure 2C). Importantly, we also observed that IFNγ signaling in IMs (as read out by CXCL9 expression) is also rescued in *Sp140*^−/−^*Ifnar1*^−/−^ mice as compared to *Sp140*^−/−^ mice (Figure 2D).

To explore whether STAT2 and IRF9 contribute to IFNAR-dependent inhibition of IFNγ signaling *in vivo*, we employed CRISPR gene editing to generate *Sp140*^−/−^*Stat2*^−/−^ and *Sp140*^−/−^ *Irf9*^−/−^ mice, respectively. Notably, single STAT2 or IRF9-deficency was sufficient to rescue type I IFN driven *Mtb* susceptibility and restore IFNγ responsiveness (Figures 2C, D). To validate these results, we also examined NOS2 expression. Compared to CXCL9, NOS2 was mainly expressed in infected (as opposed to uninfected bystander) IMs (Figures S2B–C). Focusing on infected IMs, we observed a similar connection of type I IFN driven *Mtb* susceptibility and attenuated NOS2 expression (Figures S2D–E).

During *Mtb* infection, B6 mice express lower levels of type I IFN as compared to *Sp140*^−/−^ mice, and do not show a consistent type I IFN-driven susceptibility to *Mtb*^34,36,43–45^. Thus, if the susceptibility of *Sp140*^−/−^ mice is due to impairment of the IFNγ response by type I IFN signaling, then we reasoned that we should not observe such impairment in wild-type B6 mice. Indeed, *Ifnar1*-deficiency had no effect on CXCL9 nor NOS2 expression on a B6 background (Figures 2E, S2F). If anything, we detected lower CXCL9 levels in B6.*Ifnar1*^−/−^ mice as compared to B6 IFNAR-sufficient mice.

Overall, our findings indicate that the elevated and/or sustained levels of type I IFNs in *Sp140*^−/−^ mice impair the IFNγ response to *Mtb in vivo*, and that disruption of type I IFN signaling through STAT2 or IRF9 deficiency is sufficient to overcome this effect *in vivo*.

### Type I IFNs impair IFNγ-responsiveness and *Mtb* control cell-intrinsically

**Figure 3.**
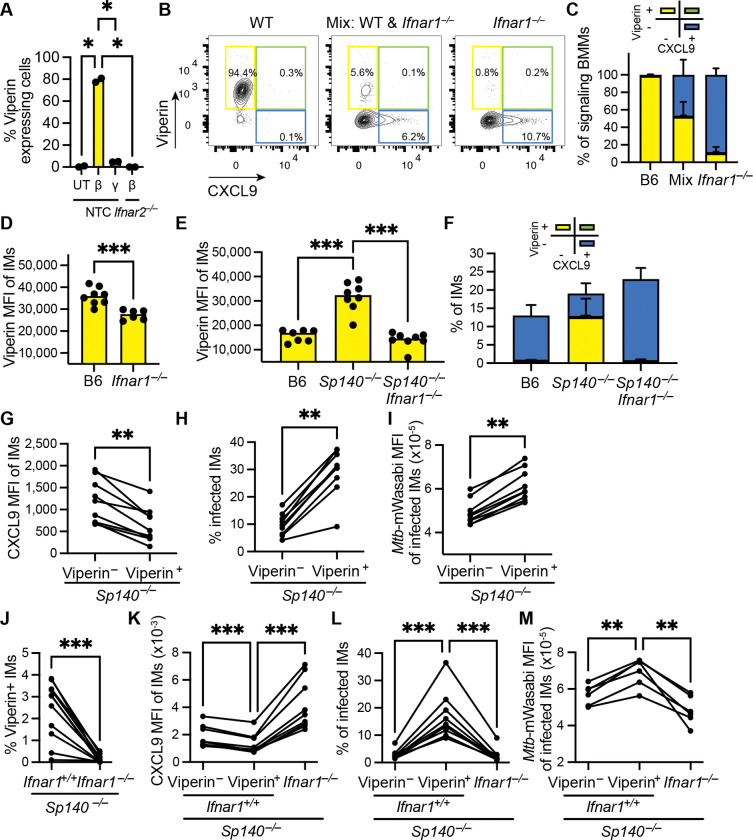
Type I IFNs impair IFNγ-responsiveness and *Mtb* control cell-intrinsically. **(A)** Quantification of Viperin-expressing cells of Non-Target Control (NTC) or *Ifnar2*^−/−^ BMMs upon IFNβ or IFNγ. UT = Untreated. **(B)** Representative flow cytometry plots and **(C)** quantification of CXCL9 and/or Viperin expressing cells from pure WT (left), *Ifnar1*^−/−^ (right) or mixed WT *Ifnar1*^−/−^ (middle) BMM culture upon exposure to both IFNβ and IFNγ. **(D)** MFI of Viperin staining in IMs from *Mtb*-infected B6 and B6.*Ifnar1*^−/−^ mice at day 25–26 pi. **(E)** MFI of Viperin staining in IMs and **(F)** quantification of CXCL9 and/or Viperin expressing IMs from *Mtb*-infected B6, *Sp140*^−/−^ and *Sp140*^−/−^*Ifnar1*^−/−^ mice at day 28–29 pi. **(G)** Flow cytometry-based comparison of Viperin-positive and -negative IMs from *Mtb*-infected *Sp140*^−/−^ mice at day 28–29 pi showing CXCL9 MFI **(H)** percentage infected IMs and **(I)** *Mtb*-mWasabi MFI. **(J-M)** Analysis of *Mtb*-infected mixed *Sp140*^−/−^ bone marrow chimeras containing IFNAR-proficient (CD45.1) and -deficient cells (CD45.2) at day 25–26 pi. **(J)** MFI of Viperin staining in *Ifnar1*^+/+^ and *Ifnar*^−/−^ IMs. **(K)** Comparison of *Ifnar1*^+/+^ IMs (negative or positive for Viperin) and *Ifnar*^−/−^ IMs (negative for Viperin, see (J)) in terms of CXCL9 MFI, **(L)** percentage infected IMs and **(M)** *Mtb*-mWasabi MFI. Statistical significance was calculated in (A, E) with Brown-Forsythe/Welch ANOVA test and in (D) with Welch’s t-test, both with Dunnett’s T3 multiple comparisons test, in (G-J) Paired t-test, in (K-M) RM one-way ANOVA with Sidak’s multiple comparison test. *p < 0.05, **p < 0.01, ***p < 0.001, ns = not significant.

Type I IFN signaling has been suggested to impair the IFNγ response cell-intrinsically by downregulating IFNγ receptor levels or cell-extrinsically by upregulating the immunosuppressive cytokine IL-10^53–56^. Therefore, we explored in our *in vitro* system if type I IFN signaling impairs the IFNγ response in a cell-intrinsic or -extrinsic manner. First, we sought to identify ISGs that could identify type I IFN-responsive cells by flow cytometry. *Rsad2* (encoding Viperin) was abundantly expressed upon IFNβ exposure at the mRNA (Figure S1F) and protein levels (Figure 3A). Importantly, expression of Viperin protein was specific to type I IFN signaling (and not observed upon IFNγ exposure) and was dependent on IFNAR (Figure 3A). We then mixed IFNAR-proficient and -deficient BMMs and exposed them to IFNβ and IFNγ to determine whether impairment of the IFNγ response is intrinsic to IFNβ-responsive (Viperin^+^) cells. Strikingly, expression of CXCL9 and Viperin was mutually exclusive (Figures 3B-C, S3A), indicating that the presence of IFNβ-responsive cells does not affect IFNγ signaling by IFNβ-non-responsive cells. These data imply that in our *in vitro* system, type I IFN impairs the IFNγ response cell-intrinsically and that IFNβ-responsive cells do not produce a soluble mediator that is sufficient to impair IFNγ signaling (Figures 3B-C, S3A).

We then examined Viperin and CXCL9 expression by IMs in *Mtb*-infected mice. Mice were infected with an *Mtb* strain expressing a fluorescent reporter, the intensity of which was previously validated to correlate with *Mtb* CFU *in vivo*^32^. As expected, Viperin staining was IFNAR-dependent in *Mtb*-infected B6 and *Sp140*^−/−^ mice (Figures 3D-E). Given that, we infected B6, *Sp140*^−/−^ and *Sp140*^−/−^*Ifnar1*^−/−^ mice and performed intracellular staining for CXCL9 and Viperin. In line with the *in vitro* results, expression of CXCL9 and Viperin in IMs was mutually exclusive (Figures 3F, S3B). Moreover, in *Sp140*^−/−^ mice, Viperin-positive IMs not only expressed reduced levels of CXCL9 but were also more frequently infected and had elevated levels of *Mtb* (Figures 3G-I). These data suggested a direct connection between elevated type I IFN-signaling, reduced IFNγ-responsiveness, and elevated *Mtb* burdens within the same cell. However, a caveat of these experiments is that the results could be confounded by the different *Mtb* burdens in *Sp140*^−/−^ versus *Sp140*^−/−^*Ifnar1*^−/−^ mice.

As a controlled genetic test of whether type I IFN signaling cell-intrinsically impairs IFNγ response and renders IMs susceptible to *Mtb*, we established mixed bone marrow chimeras (BMCs) in which CD45.1 IFNAR-proficient and CD45.2 IFNAR-deficient *Sp140*^−/−^ bone marrow cells were used to reconstitute *Sp140*^−/−^ recipients. As a control we also generated chimeric mice reconstituted with a mix of CD45.1 and CD45.2 bone marrow cells, both of which were IFNAR-proficient (Figures S3C–F). Mice were infected with *Mtb* >8 weeks after hematopoietic reconstitution. In these chimeras, IFNAR-proficient and IFNAR-deficient cells respond to *Mtb* and IFNγ in the same inflammatory environment. Importantly, Viperin staining revealed that only a minor fraction (~1–4%) of *Ifnar1*^+/+^ IMs actively respond to type I IFN (Figure 3J, control in Figure S3C). Due to the rapid kinetics of Viperin induction and turnover, the expression of Viperin is transient (Figure S1F), and thus Viperin expression may fail to identify all the cells that have sensed type I IFNs (see below). Nevertheless, we consistently found decreased CXCL9 levels and elevated *Mtb* burdens in these few Viperin expressing *Ifnar1*^+/+^ IMs compared to *Ifnar1*^−/−^ IMs (which fail to induce Viperin; Figure 3J) or to Viperin-negative *Ifnar1*^+/+^ cells (Figures 3K-M). Although many *Ifnar1*^+/+^ cells in infected mice did not respond to IFNβ and were Viperin-negative (Figure 3J), it was still possible to detect increased CXCL9 expression and decreased *Mtb* infection by percentage and MFI in the total *Ifnar1*^+/+^ cell population (Figures S3D–F).

In summary, these observations suggest that in IMs, type I IFN predominantly impairs IFNγ response in a cell intrinsic manner to promote *Mtb* susceptibility.

### Sustained strong type I IFN-signaling impairs the response to IFNγ and precede*s Mtb su*sceptibility

**Figure 4.**
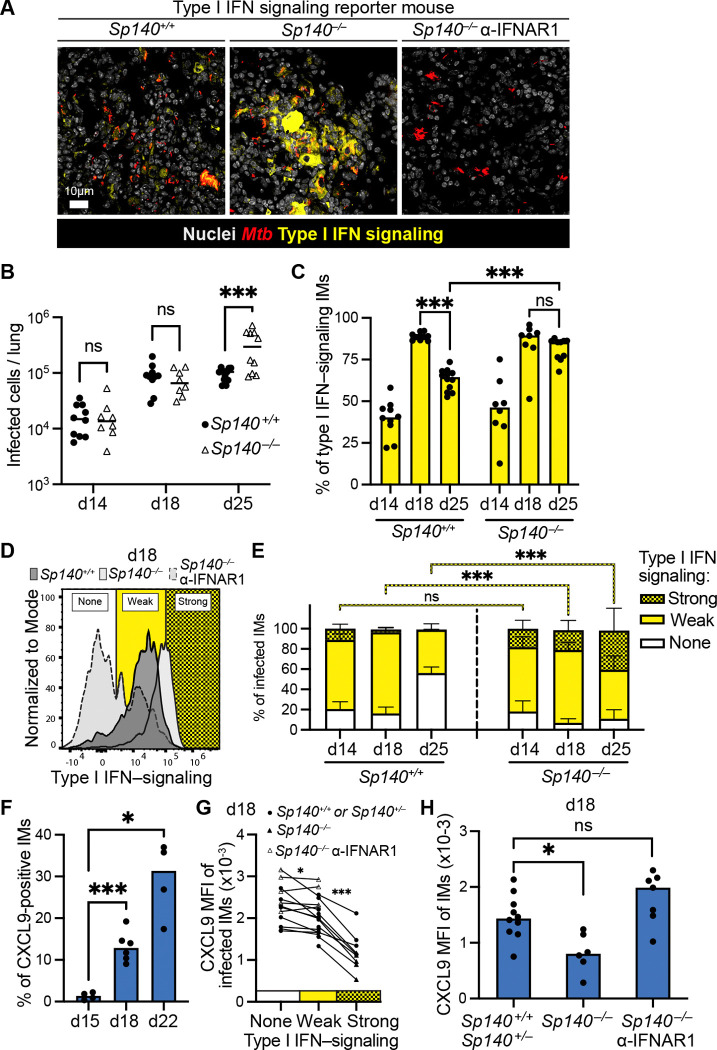
Sustained and strong type I IFN–signaling impairs the response to IFNγ and precedes *Mtb* susceptibility **(A)** Representative micrograph of lung tissue from *Mtb*-infected *Sp140*^+/+^ and *Sp140*^−/−^ Mx1-GFP reporter mice without or with administration of anti-IFNAR1 antibodies at day 18 pi. **(B)** Quantification of *Mtb*-infected cells and **(C)** Mx1-GFP-expressing IMs from lung tissue of *Sp140*^+/+^ and *Sp140*^−/−^ mice at day 14, 18 and 25 pi. **(D)** Representative flow cytometry histogram of Mx1-GFP expression of infected IMs from lung tissue of *Sp140*^+/+^ and *Sp140*^−/−^ mice without or with administration of anti-IFNAR1 antibodies at day 18 pi. Different levels type I IFN signaling are indicated. **(E)** Quantification of percentage of infected IMs with different levels of type I IFN signaling (as indicated in (D)) from lung tissue of *Sp140*^+/+^ and *Sp140*^−/−^ mice at day 14, 18 and 25 pi. **(F)** Percentage of CXCL9 expressing IMs from lung tissue of *Mtb*-infected B6 mice at day 15, 18 and 22. **(G)** Comparison of CXCL9 MFI between none, weak and strong type I IFN signaling IMs (as indicated in (D)) from lung tissue of *Sp140*^+/+^ or *Sp140*^+/−^ and *Sp140*^−/−^ mice without or with administration of anti-IFNAR1 antibodies at day 18 pi. **(H)** CXCL9 MFI of IMs from lung tissue of *Sp140*^+/+^ or *Sp140*^+/−^ and *Sp140*^−/−^ mice without or with administration of anti-IFNAR1 antibodies at day 18 pi. Statistical significance was calculated in (B, C, E) with Two-way ANOVA and Sidak’s multiple comparison test, in (F, H) with Brown-Forsythe and Welch ANOVA test and Dunnett’s T3 multiple comparisons test, in (G) Mixed-effects analysis, with the Geisser-Greenhouse correction and with Sidak’s multiple comparison test with individual variances computed for each comparison. *p < 0.05, **p < 0.01, ***p < 0.001, ns = not significant.

Although Viperin expression marked some type I IFN-responsive cells, Viperin expression is transient (Figure S1F) and is lost before the repressive effects of type I IFN on IFNγ signaling have resolved. Thus, Viperin expression likely underreports type I IFN signaling. As a more sensitive reporter for type I IFN signaling, we took advantage of a previously described type I IFN signaling reporter mouse^65^ and crossed it to our *Sp140*^−/−^ mice. In this reporter mouse, green fluorescent protein (GFP) is expressed under the control of the native promoter of Mx1, a type I IFN-induced gene that is transcribed but does not encode a functional protein in mice. The long half-life of GFP allows for sensitive and sustained detection of type I IFN-responsive cells. We confirmed in BMMs that GFP is strongly induced upon IFNβ (Figure S4A) and that on day 18 post-*Mtb* infection, we could detect type I IFN-responsive cells in lung lesions of *Sp140*^−/−^ mice (Figures 4A, S4B), with much weaker reporter expression in B6 mice. Importantly, administration of anti-IFNAR1 antibody, previously shown to rescue the susceptibility of *Sp140*^−/−^ mice, reduced Mx1-GFP reporter expression below the levels in B6, indicating that the Mx1-GFP reporter is specific for type I IFN signaling *in vivo* in *Mtb*-infected mice.

We then analyzed the lungs of B6 and *Sp140*^−/−^ Mx1-GFP reporter mice at days 14-, 18- and 25 pi. Interestingly, while type I IFN signaling was detected in IMs as early as 14-days pi in both B6 and *Sp140*^−/−^ mice, the enhanced susceptibility of *Sp140*^−/−^ mice to *Mtb* only became apparent at day 25 pi (Figure 4B-C). Surprisingly, at day 14 and 18 pi, the frequency of type I IFN sensing IMs was similar across genotypes. However, by day 25 pi, the prevalence of type I IFN signaling IMs decreased in B6 mice, while in *Sp140*^−/−^ mice it remained high (Figure 4C). Importantly, the reporter mouse line allowed us to define three levels of type I IFN signaling: none, weak and strong (Figure 4D, weak is expression levels greater than background levels, whereas strong is expression levels greater than that seen in infected B6 mice at day 18 pi). At early time points (i.e., day 14 pi), the strength of IFN signaling in B6 and *Sp140*^−/−^ mice was similar (Figure 4E). However, by day 18 pi, elevated numbers of IMs responding strongly to type I IFN were present in *Sp140*^−/−^ mice, as compared to B6 mice (Figure 4E). While in both genotypes the numbers of weak type I IFN signaling IMs dropped over time, cells exhibiting strong type I IFN signaling increased in number in *Sp140*^−/−^ mice at day 25 pi, but largely disappeared in B6 counterparts (Figure 4E). These observations suggest that in contrast to B6, a substantial subset of IMs from *Sp140*^−/−^ mice exhibit a stronger and more sustained type I IFN response than in B6 mice. Moreover the sustained strong type I IFN responsiveness of IMs in *Sp140*^−/−^ mice precedes the appearance of elevated *Mtb* burdens in these mice.

In contrast to type I IFN signaling, the IFNγ response as assessed by CXCL9 expression was only apparent as early as 18-days pi (Figure 4F), which most likely co-occurs with the arrival of *Mtb*-specific T cells^66^. At this timepoint, we found that cells mounting a strong type I IFN response exhibited markedly decreased CXCL9 expression as compared to cells responding weakly or undetectably to type I IFNs (Figure 4G). The effect of strong type I IFN signaling on CXCL9 induction was evident in both B6 and *Sp140*^−/−^ mice; however, because there were considerably more cells responding strongly to type I IFNs in *Sp140*^−/−^ mice, these mice exhibited an overall decrease in the levels of CXCL9 expression by IMs in the lung, as compared to B6 lungs (Figure 4H). Importantly, at this timepoint, CFU burdens were comparable between B6 and *Sp140*^−/−^ mice with and without anti-IFNAR1 antibody treatment (Figure S4C). Thus, type I IFN-dependent defects in IFNγ signaling preceded loss of bacterial control.

Together, our results demonstrate that type I IFNs are induced at early timepoints during *Mtb* infection, preceding the onset of the IFNγ response. In B6 mice, the type I IFN response is weak and transient; thus, the responsiveness to IFNγ at later timepoints is unimpaired, and bacterial burdens are controlled. However, in *Sp140*^−/−^ mice, many cells respond strongly and persistently to type I IFN signaling, and these cells exhibit defective responsiveness to IFNγ and fail to restrain *Mtb* replication.

### RESIST stabilizes and strengthens type I IFN responses, impairs IFNγ responses, and promotes susceptibility to *Mtb*

**Figure 5.**
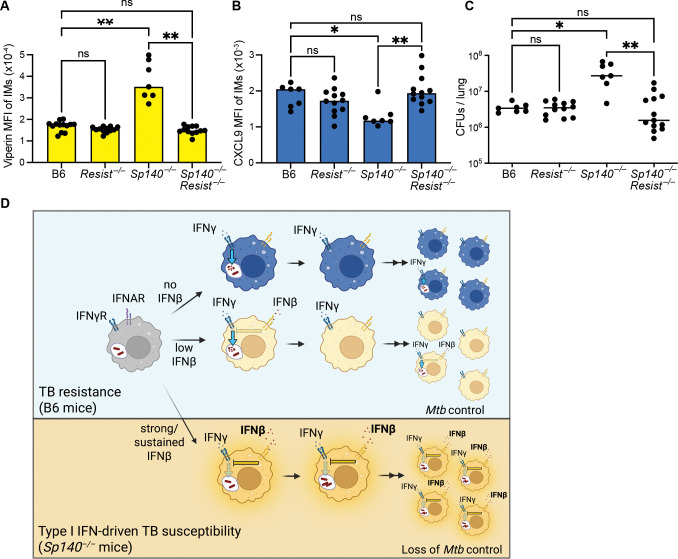
RESIST stabilizes and strengthens type I IFN responses, impairs IFNγ responses, and promotes susceptibility to *Mtb*. **(A)** Viperin MFI **(B)** CXCL9 MFI and **(C)** CFUs of IMs from lung tissue of B6, *Resist*^−/−^, *Sp140*^−/−^ and *Sp140*^−/−^*Resist*^−/−^ mice at day 26–28 pi. **(D)** Model how sustained and strong type I IFN signaling impairs IFNγ responses and causes *Mtb* susceptibility. Illustration created with BioRender.com. Statistical analysis was calculated in (A-B) with Brown-Forsythe/Welch ANOVA test with Dunnett’s T3 multiple comparisons test and in (C) with Kruskal-Wallis test and Dunn’s multiple comparison test. *p < 0.05, **p < 0.01, ***p < 0.001, ns = not significant.

Genetic or antibody-mediated blockade of IFNAR signaling rescues IFNγ signaling and *Mtb* susceptibility of *Sp140*^−/−^ mice^41^ (Figures 2D, 4G). However, these conditions eliminate all type I IFN signaling and therefore do not specifically eliminate the sustained and strong type I IFN response that our data suggest is responsible for *Mtb* susceptibility. We recently discovered RESIST (encoded by *Resist1* and *Resist2*) is a direct target of SP140 repression and is a crucial positive regulator that is required for enhanced IFNβ expression in *Sp140*^−/−^ mice. In *Sp140*^−/−^ mice, RESIST is de-repressed and acts to stabilize IFNβ transcripts, thereby sustaining and strengthening type I IFN responses. To test whether this sustained and strong type I IFN signaling is the cause of type I IFN driven *Mtb* susceptibility, we tested if genetic loss of *Resist1* and *Resist2* (hereafter *Resist*^−/−^) rescues *Sp140*^−/−^ mice. Indeed, while *Resist*-deficiency had little impact in B6 mice (in which Resist is not normally expressed), *Sp140*^−/−^*Resist*^−/−^ mice were fully rescued for appropriate type I IFN signaling as compared to *Sp140*^−/−^ mice (Figure 5A). Restoration of appropriate type I IFN signaling was also sufficient to restore the IFNγ response based on CXCL9 and NOS2 staining (Figures 5B, S5A). Consequently, RESIST-deficiency fully rescued the *Mtb* susceptibility of *Sp140*^−/−^ mice (Figure 5C). Importantly, the rescue mediated by loss of *Resist* occurred without impairment of ‘normal’ IFNAR signaling. Thus, a normal (i.e., transient and modest) type I IFN response does not impair IFNγ responsiveness or *Mtb* control. Instead, our data demonstrate that it is specifically a strong and/or sustained type I IFN response that mediates *Mtb* susceptibility (Figure 5D).

## Discussion

Type I IFNs consistently correlate with human TB disease progression^21–25^ and have been shown to cause *Mtb* susceptibility in numerous mouse models^26–35^. Previous studies have found that type I IFNs impair the protective IL-1 response via multiple mechanisms^34,36,37^. However, type I IFNs also promote TB susceptibility via IL-1-independent mechanisms^37,39,40^. Here, we provide evidence that sustained and strong (but not basal) type I IFN signaling cell-intrinsically impairs IFNγ responsiveness in *Mtb*-infected macrophages in the lung. We further show that the impairment of IFNγ responsiveness is an early and primary consequence of type I IFN signaling that renders IMs permissive for intracellular *Mtb* replication, initiating the loss of bacterial control and disease susceptibility.

In our study, we have taken advantage of SP140-deficient mice, which we previously showed to be a physiological relevant congenic mouse model that mirrors key aspects of human clinical TB^32,41^. A major advantage of *Sp140*^−/−^ mice over other models of type I IFN-mediated susceptibility is that elevated type I IFN signaling arises endogenously during *Mtb* infection, without the need for artificial induction with a synthetic ligand or viral-coinfections. Notably, data derived from viral coinfection models are often difficult to interpret, as observed phenotypes may reflect differences in viral burden rather than type I IFN signaling per se. Unlike other *Mtb*-susceptible mouse strains (e.g., C3HeB/FeJ (“Kramnik”) or 129 mice), *Sp140*^−/−^ mice are on a pure C57BL/6J background, allowing for controlled experimental crosses to the many other genetically engineered strains that have been generated on the same background. The primary effect of SP140-deficiency appears to be a relatively selective increase in the levels of IFNβ, and importantly, the elevated *Mtb* CFU burdens of *Sp140*^−/−^ mice are fully rescued by crossing to *Ifnar1*^−/−^ mice. We recently reported that the mechanistic basis for the enhancement of IFNβ levels in *Sp140*^−/−^ mice is due to the selective de-repression of RESIST, a positive and selective positive regulator of IFNβ mRNA stability^42^. Thus *Sp140*^−/−^ mice represent a well-characterized, physiological, and specific model for the mechanistic dissection of type I IFN-driven tuberculosis disease.

Previous *in vitro* studies have shown that type I IFNs can impair the induction of selected IFNγ-responsive genes^50–53^. However, many ISGs are induced by both type I IFNs and IFNγ, complicating efforts to clearly distinguish the two transcriptional responses and to determine whether type I IFNs broadly suppress IFNγ responses. Here, we overcome this limitation by performing transcriptome analyses using a curated set of 21 “IFNγ signature” genes that we previously defined to be preferentially induced by IFNγ over type I IFNs, based on *in vitro* stimulations and *Mtb*-infected mice^32^ (Figure 1). We show that IFNβ pre-exposure potently impairs the induction of the majority of IFNγ signature genes. Importantly, this effect extends well beyond this defined gene set, suppressing more than 90% of genes that are induced at least two-fold more strongly by IFNγ than by IFNβ. Importantly, we also find that the impairment of IFNγ signaling persists well after active type I IFN signaling has subsided, requires the IFNAR-dependent signal transducers STAT2 and IRF9, and is conserved in both mouse and human primary macrophages.

Notably, three IFNγ-specific ISGs that were resistant to IFNβ-mediated suppression belonged to the guanylate-binding protein (GBP) family (Figure 1D). Two of these genes, *GBP2B* and *GBP10*, are encoded within a chromosome 3 GBP cluster previously shown to mediate IFNγ-dependent protection against multiple bacterial infections^67–69^. In contrast, recent work demonstrated that chromosome 3 GBPs are dispensable for protection during *Mtb* infection^70^. Thus, while chromosome 3 GBPs can confer IFNγ-dependent protection in other bacterial infections, and are resistant to type I IFN-mediated impairment, protective immunity to *Mtb* relies predominantly on IFNγ-induced genes that are highly sensitive to suppression by type I IFNs. Our results may thus help explain why type I IFN-driven susceptibility is particularly pronounced in TB.

In human *Mycobacterium leprae* infections, elevated type I IFN signatures correlate with impaired IFNγ responses and poor clinical outcomes^54^. For *Mtb*, we recently reported impaired IFNγ responses in *Sp140*^−/−^ mice^32^. However, a causal relationship had not been established. Based on our *in vitro* stimulation experiments and genetic evidence in mice, we identified CXCL9 as a robust marker of IFNγ responsiveness in IMs during *Mtb* infection (Figures 1, 2). We therefore infected B6, *Sp140*^−/−^, and *Sp140*^−/−^*Ifnar1*^−/−^ mice with *Mtb* and analyzed responses at day 25–28 post infection, demonstrating that type I IFN signaling impairs IFNγ responsiveness *in vivo* (Figure 2). Notably, at this time point (also used in prior studies^32^) bacterial burdens had already diverged between genotypes, complicating discrimination between a direct inhibitory effect of IFNAR signaling on IFNγ responses and an indirect secondary consequence of increased bacterial loads. To resolve this, we performed a time-course analysis (Figure 4), which revealed a clear temporal progression in which early type I IFN signaling precedes and suppresses the later-emerging IFNγ response, ultimately resulting in elevated bacterial burdens.

To precisely identify type I IFN-responsive cells at the single cell level, we established Viperin expression as a reliable marker of active type I IFN signaling. Combined Viperin and CXCL9 staining in mixed *Sp140*^−/−^ bone marrow chimeras containing IFNAR-proficient and -deficient cells demonstrated that type I IFNs impair IFNγ responsiveness predominantly in a cell-intrinsic manner (Figure 3).

The mechanism by which type I IFNs impair IFNγ signaling has been studied extensively, but a single dominant mechanism has yet to emerge. It has been proposed that type I IFNs suppress IFNγ receptor expression^53,55^. Consistent with this, our RNA-seq analysis revealed a twofold reduction in *Ifngr1* expression following IFNβ stimulation, and we previously reported reduced IFNγ receptor expression in SP140-deficient mice^32^. However, whether reduced receptor expression alone accounts for the observed impairment remains unclear. Additional, potentially redundant mechanisms are likely to contribute. For example, the shared signal transducer STAT1, which operates downstream of both IFNAR and IFNγR, may represent a limiting factor under conditions of sustained type I IFN signaling^71^. Moreover, our transcriptomic data show robust induction of SOCS1, a well-established negative regulator of IFNγ signaling, following IFNβ exposure. Beyond these mechanisms, type I IFNs induce a broad antiviral response that includes ISGs known to suppress gene expression at both the transcriptional and translational levels, such as PKR-mediated eIF2α phosphorylation, IFIT-dependent inhibition of translation initiation, and OAS–RNase L–mediated RNA degradation^72^. Thus, it is reasonable to speculate that type I IFN–mediated suppression of IFNγ responsiveness is multifactorial, arising from the combined action of receptor-level regulation, shared signaling constraints, negative feedback pathways, and global repression of gene expression.

A major unresolved question has been why type I IFNs promote TB pathogenesis in certain mouse strains (e.g., C3HeB/FeJ^34,73^ or 129^74^), but not in B6 mice. This has been puzzling because B6 mice are fully capable of mounting a robust type I IFN response, and indeed, are generally highly resistant to viral infections^75–77^. Previous data have shown that type I IFNs are expressed at higher levels in susceptible mouse strains during *Mtb* infection, but since *Mtb* itself induces type I IFNs^45,78^, it has been difficult to determine if mice are susceptible due to higher interferon levels, or if higher bacterial burdens in susceptible mice are what cause the increased interferon responses. Prior experiments have shown that deleting *Ifnar* in susceptible mice^34,74^ restores resistance, but these experiments do not address whether enhanced IFN levels drive susceptibility, or vice-versa, because *Ifnar* deletion eliminates all type I IFN signaling and does not selectively eliminate the elevated levels while maintaining basal interferon responses. To gain insight into the quantitative dynamics by which type I IFN signaling impairs IFNγ responses and promotes *Mtb* susceptibility, we crossed *Sp140*^−/−^ mice to the sensitive type I IFN reporter line Mx1-GFP, in which type I IFN signaling induces expression of a stabilized GFP^65^. With these mice, we show that IFNγ responsiveness is not determined simply by the presence or absence of type I IFN signaling but instead depends critically on the magnitude and/or duration of the response (Figure 4). Specifically, strong and/or sustained type I IFN signaling suppresses IFNγ responsiveness and promotes *Mtb* susceptibility. Importantly, our time course analysis shows that the enhanced type I IFN response in *Sp140*^−/−^ mice versus wild-type mice occurs prior to the divergence in bacterial burdens, and is thus not a consequence of elevated bacterial burdens, but is instead an important driver of *Mtb* susceptibility. This conclusion is supported not only by data from the Mx1-GFP reporter mice, but also from independent genetic experiments (Figure 5) in which we deleted the *Resist* locus that we previously showed encodes a positive regulator of type I IFN production^42^. *Resist* deletion had no detectable effect in the B6 background (in which it is not normally expressed) and did not affect basal type I IFN levels. However, in *Sp140*^−/−^ mice, RESIST-deficiency restored an appropriately transient type I IFN response, thereby permitting IFNγ-dependent restriction of *Mtb* comparable to that observed in B6 mice.

Our findings emphasize the key point that IFNγ production does not necessarily translate into effective IFNγ signaling. Instead, IFNγ responsiveness is shaped by the surrounding cytokine environment, particularly by the presence of type I IFNs, and this must be considered when evaluating IFNγ function. Accordingly, assessment of IFNγ activity requires measurement of downstream responses, rather than IFNγ levels alone. Here, we provide robust markers to reliably quantify IFNγ responsiveness and offer a clear example in the context of TB of how this distinction is critical. Together, our findings might provide an answer for why induction of IFNγ or IFNγ-producing CD4+ T cells^11–15^ is not always sufficient to confer protection against *Mtb* or other bacterial pathogens in which type I IFN-driven susceptibility is observed. We also reveal potential strategies for enhancing IFNγ-mediated immunity that could lead to more effective host directed therapies.

## Materials & Methods

### Mice

Mice were maintained in accordance with the regulatory standards of the University of California Berkeley Institutional Animal Care and Use Committee under specific pathogen-free conditions. In all experiments mice were age- and sex-matched and were 8–18 weeks old at the start of the infections. Every experiment included female and male mice. Mouse lines used are C57BL/6J (B6), B6.129S2-*Ifnar1*^tm1Agt/Mmjax^ (*Ifnar1*−/−), B6.129S7-*Ifngr1*tm1Agt/J (*Ifngr1*−/−), C57BL/6J-*Ptprc*em6Lutzy/J (CD45.1) and B6.Cg-*Mx1*^tm1.1Agsa/J^ (*Mx1-gfp*) that were purchased from Jackson Laboratories. *Sp140*^−/−^ previously made^41^ was crossed in house to *Ifnar1*^−/−^, CD45.1 and *Mx1-gfp* to generate *Sp140*^−/−^ *Ifnar1*^−/−^, *Sp140*^−/−^ CD45.1 and *Sp140*^−/−^*Mx1-gfp*, respectively. *Sp140*^−/−^*Resist*^−/−^ were previously described^42^ and backcrossed in house to B6 to generate *Resist*^−/−^.

### Generation of *Sp140*^−/−^*Irf9*^−/−^ and *Sp140*^−/−^*Stat2*^−/−^ mice

*Sp140*^−/−^Irf9^−/−^ and *Sp140*^−/−^Stat2^−/−^ mice were generated by electroporation of *Sp140*^−/−^ zygotes with Cas9 and sgRNA UACGCUGCACCCGAAAGCUG and AGUGGUCCCACUGGUUCAGU, respectively. Founders were genotyped and backcrossed to *Sp140*^−/−^ mice, and progeny with matching alleles were further bred. Genotyping was performed by sequencing of PCR product with the primer pairs CAGGGGTTTGCAAGTTGTTG, AGACATGGTTGGTTCTACTTTCT for *Irf9* and GGCTCATCTGATTTCAGGCC, CCTCTCAGGTGACACACAAC for *Stat2*. Established knock-out mouse lines had a 20, 17 base pair deletion in *Irf9* exon 3, *Stat2* exon 3, respectively.

### Mouse *Mtb* infections, *in vivo* antibody blockade, tissue processing for CFU and flow cytometry analysis

*Mtb* Erdman strain including the ones constitutively expressing either mWasabi or mCherry have previously been described^32^. Inoculum was prepared from a frozen stock and diluted in 9ml sterile PBS at an of ca. OD of 0.002. Mice were inserted into an aerosolizer device (Glas-Col, Terre Haute, IN) and infected through the aerosol route at a low dose of ca. 20–100 CFUs. Infectious dose was verified from 2–3 mice at day 1 post infection. For IFNAR1 blockage *in vivo* 500ug anti-IFNAR1 antibody (bioXcell, MAR1–5A3) per mouse was injected intraperitoneal every other day starting at day 7 pi. Mice were sacrificed at indicated time points post infections and the complete lungs were harvested into a GentleMACS C tube (Miltenyi Biotec) with 2ml digestion media consisting of 2ml RPMI media (Gibco) with 30ug/ml DNase I (Roche), 70ug/ml Liberase TM (Roche) and Brefeldin A (BioLegend). Lungs were cut into large pieces using the program lung_01 on GentleMACS device (Miltenyi Biotec) before incubating at 37°C for 30 minutes. Lungs were homogenized with program Lung_2 on the GentleMacs device and digestions was stopped by adding 2ml of PBS including 20% Newborn Calf Serum (Thermo Fisher Scientific). Lung homogenate was filter through a 70um SmartStrainers (Miltenyi Biotec) into 15ml falcon tube. From this single cell suspension 100ul was saved for CFU plating assay, whereas the rest centrifuged at 1,600 rpm for 8 minutes at 4°C. Cell pellet was resuspended in FACS buffer and used for flow cytometry analysis.

### CFU plating

7H11 plates supplemented with 10% BD BBL^™^ Middlebrook OADC Enrichment (Fisher) and 0.5% glycerol were prepared ahead of time and stored at 4°C. Lung homogenates were serial diluted in PBS and 50ul of appropriate dilutions were plated. After 3 weeks incubation at 37°C, CFUs were enumerated to back calculate CFUs per lung.

### Flow cytometry of lung homogenate

The following fluorophore-coupled antibodies were used accordingly together with fixable viability dye (Ghost Dye^™^ Violet 780; Tonbo Biosciences), TruStain FcX PLUS (S17011E, BioLegend), Super Bright Complete Staining Buffer (Thermo Fisher Scientific) and True-Stain Monocyte Blocker (BioLegend) to prepare a Master Mix and stain the single cells suspension from above: BV421-coupled MHCII (M5/114.15.2, BioLegend), BV480-coupled B220 (RA3–6B2, BD Biosciences), BV480-coupled CD90.2 (53–2.1, BD Biosciences), BV605-coupled CD64 (X54–5/7.1, BioLegend), BV711-coupled CD11b (M1/70, BioLegend), BV785-coupled Ly6C (HK1.4, BioLegend), PE-Cy7-coupled MerTK (DS5MMER, Thermo Fisher Scientific), APC-R700-coupled Siglec F (E50–2440, BD Biosciences), BUV496-coupled CD45 (30-F11, BD Biosciences), BUV563-coupled Ly6G (1A8, BD Biosciences), BUV737-coupled CD11c (HL3, BD Biosciences), BV421-coupled CD45.1 (A20, BioLegend), BUV496-coupled CD45.2 (104, BD Biosciences). Staining was performed at room temperature for >30 minutes before washing the cells three times with FACS buffer. Stained cells were fixed with Cytofix/Cytoperm (BD Biosciences) for >30 minutes at room temperature before retrieved from BSL3 facility. Fixed cells were permeabilized by washing cells four times with Permeabilization Buffer (Invitrogen) before performing intracellular staining using PE-coupled Viperin (MaP.VIP, BD Biosciences), AF647-coupled CXCL9 (MIG-2F5.5, BioLegend) and BUV395-coupled NOS2 (CXNFT, Invitrogen). Cells were run on an Aurora (Cytek) flow cytometer and analyzed with Flowjo version 10 (BD Biosciences).

### Microscopy

For microscopy the middle lobe was harvested and directly fixed with Cytofix/Cytoperm (BD Biosciences) diluted in PBS (1:2) for >24h at 4°C before retrieving from BSL3 laboratory. Fixed lung tissues were washed with PBS and dehydrated for >12h at 4°C in PBS containing 20% sucrose. Lung tissues were embedded in O.C.T. (Tissue-Tek) and stored at −80°C. 10um sections were prepared using a Leica CM3050S Cryotome and mounted on Superfrost Plus Microscope Slides (Fisher). Sections were rehydrated with PBS, permeabilized with PBS containing 0.5% Tx-100 and blocked with 10% Normal Goat Serum (Vector Laboratories) before staining with DAPI (Sigma) and fluorophore-coupled antibodies. Stained tissue was covered with a cover slip and VectaShield HardSet (Vector Laboratories). A Zeiss LSM710 confocal microscope and FIJI software was used for image analysis.

### Bone Marrow Chimeras

From donor mice, bones (femurs and tibias) were harvested, sterilized in 70% EtOH and bone marrow was isolated by flushing the bones with cold PBS using a syringe and filtering through a 70um cell strainer. Cells were once washed and resuspended in PBS for injection. For mixed bone marrow chimeras, bone marrow cells from different genotypes were mixed in a 1:1 ratio. Recipient mice were lethally irradiated two times within 12–20 hours with a Precision X-Rad320 X833 Ray irradiator (North Branford, CT). Mice received 2–5 Mio bone marrow cells in 200ul PBS by retro-orbital injection. Mice were housed for >8 weeks to allow hematopoietic reconstitution prior Mtb infection.

### Bone marrow macrophages (BMMs) establishment and cytokine exposures for flow cytometry analysis

Bones (femurs and tibias) from B6 or *Ifnar1*^−/−^ mice were harvested and sterilized in 70% EtOH. Bone marrow was isolated by flushing the bones with ice-cold BMM media consisting of DMEM supplemented with 10% fetal bovine serum (FBS), 10% MCSF (generated from 3T3 cells), GlutMax, 10mM HEPES and Pen-Strep (Thermo). Isolated bone marrow was filtered through a 70um cell strainer, centrifuged at 4°C for 5 min, 600G, resuspended in BMM media and seeded into 6–8 15cm non-treated petri dishes. Cells were incubated for 7 days to differentiate into BMMs with adding 50% BMM media on day 3 before transferring cells into the appropriate multi-well format using a cell scraper. After 2 days of resting, BMMs were exposed to cytokines according to the experimental setup. For exposures, cytokines expressed in either HEK293 or CHO cells were used. Specifically, if not other stated in figures or figure legends, 2ng/ml mouse IFNβ (581304, BioLegend), 50ng/ml mouse IFNγ (ab259378, Abcam), 10ng/ml mouse TNF (ab259411, Abcam), 2ng/ml human IFNβ (300–02BC, Thermo), 50ng/ml human IFNγ (ab259377, Abcam). TLR agonist used were 50ng/ml Pam3CSK4 (Invivogen, tlrl-pms) and 10ng/ml LPS (Invivogen, tlrl-3pelps).

### Flow cytometry of cytokine exposed macrophages

Brefeldin A was added to the BMM media for the last 4 hour of cytokine exposure. BMM media was removed, and BMMs were incubated for 20min with pre-warmed PBS with 4mM EDTA. Detached BMMs were transferred and washed once in PBS prior staining with fixable viability dye (Ghost Dye^™^ Violet 780; Tonbo Biosciences). After 30min incubation at room temperature, cells were washed three times with PBS and fixed with IC Fixation Buffer (eBioscience) for 15 minutes at room temperature. Cells were washed and fixed four times with Permeabilization Buffer (Invitrogen) before performing intracellular staining using PE-coupled Viperin (MaP.VIP, BD Biosciences), AF647-coupled CXCL9 (MIG-2F5.5, BioLegend) and AF488-coupled NOS2 (CXNFT, eBiosciences). Cells were run on a Fortessa (BD Biosciences) flow cytometer and analyzed with Flowjo version 10 (BD Biosciences).

### Cas9-ribonucleoprotein (RNP) mediated gene disruption in BMMs

Gene disruption in BMMs was performed with Cas9-ribonucleoprotein (RNP) electroporation at day 5 post seeding as described previously^79^. In brief, Cas9 2 NLS nuclease (Synthego) was pre-incubated with gRNAs (Synthego, sgRNA EZ kits) and Alt-R Cas9 Electroporation Enhancer (IDT, 1075916) for >20min at room temperature to form Cas9-RNPs. BMMs were lifted using a cell scraper, washed in PBS and resuspended in Lonza P3 buffer (Lonza, V4XP-3032) including Supplement 1 according to manufacturer’s protocol before combining with Cas9-RNPs. Cells were electroporated with the Lonza 4D-Nucleofector Core Unit (AAF-1002B) using the program CM-137. Electroporated BMMs were recovered in BMM media and 2Mio cells were plated per 10cm non-treated petri dish and incubated at 37°. 50% fresh media was added two days post electroporation and after 4 days of recovery edited BMMs were transferred into the appropriate multi-well format for exposure assays. The following gRNA sequences were used: *Ifnar2*: CAGACGGUGUGAUAGUCUCU & CAAAGACGAAAAUCUGACGA, *Stat2*: AGUGGUCCCACUGGUUCAGU, *Irf9*: UACGCUGCACCCGAAAGCUG & GUUGUAAACCACUCAGACAG.

### Human macrophages

THP-1 cells (ATCC GP2–293 cells, Clontech) were maintained in RPMI including 10% FBS, GlutMax and Pen-Strep (complete RPMI). THP-1 were differentiated by adding 100 ng/mL phorbol myristate acetate (PMA, Invivogen, tlrl-pma) for 48 hr followed by 36h rest before used for exposure assays. Cryopreserved negatively selected primary human monocytes were purchased from AllCells and differentiated for 6 days in complete RPMI supplemented with 50 ng/ml human M-CSF (PeproTech, 300–25). Cells were lifted with trypsin and transferred into a 96-well format for exposures.

### Bulk RNA-seq sample preparation and analysis

For exposures 10ng/ml mouse IFNβ (581304, BioLegend), 10ng/ml mouse IFNγ (ab259378, Abcam) was used. Upon cytokine exposure, BMMs were lysed with TRK lysis buffer (Omega Bio-Tek) including 2-mercaptoethanol (Thermo Fisher Scientific). Total RNA isolation was performed using the E.Z.N.A Total RNA Kit I (Omega Bio-Tek) with a DNase treatment on-column (Qiagen, 79254). The library preparation, sequencing, and read alignment to the mouse genome was performed by Azenta Life Sciences. Raw counts were used as input for analysis with DESeq2^80^.

### RT-qPCR analysis

Total RNA was isolated the same way as for bulk RNAseq described above. cDNA was reverse transcribed from RNA with Superscript III Reverse Transcriptase (Invitrogen, 18080093) and oligo dT18 (NEB, S1316S) in the presence of RNase inhibitors. Diluted cDNA was assessed by RT-qPCR using the Power SYBR Green PCR Master Mix (Thermo Fisher Scientific, 43–676-59) in technical duplicates. Prime-Time qPCR Primers (IDT) were used: *Cxcl9* Mm.PT.58.5726745, *Rsad2*: Mm.PT.58.11280480, Actin: Mm.PT.39a.22214843.g.

### Statistical analysis

Statistical tests to determine statistical significance were performed using Prism (GraphPad) software and are indicated in the figure legends. *p < 0.05, **p < 0.01, ***p < 0.001, ns = not significant.

## Supplementary Material

Supplement 1
